# Supplementary material for: Characteristics of alcohol recovery narratives: Systematic review and narrative synthesis
Source: PLoS One. 2022 May 5;17(5):e0268034. doi: 10.1371/journal.pone.0268034 (PMC9070949; doi:10.1371/journal.pone.0268034)
Supplement: Attachment — Submitted filename: Response to Reviewers.doc [file pone.0268034.s006.doc]

Department Gastroenterology
Faculty of Medicine

University of Nottingham UK

Dr Saeed Ahmed, MD
Academic Editor
PLOS ONE

Date: 21/03/2022

Dear Dr Ahmed,

I am writing to address the suggested revisions to our submitted manuscript titled, "**Characteristics of alcohol recovery narratives: systematic review and narrative** **synthesis**". Thank you to reviewers for taking the time to provide a comprehensive review.

We have attempted to address editor’s and each of the reviewer's comments, as listed below:

**Editor:**

When submitting your revision, we need you to address these additional requirements.

1.Please ensure that your manuscript meets PLOS ONE's style requirements, including those for file naming. The PLOS ONE style templates can be found at 
<https://journals.plos.org/plosone/s/file?id=wjVg/PLOSOne_formatting_sample_main_body.pdf> and 
<https://journals.plos.org/plosone/s/file?id=ba62/PLOSOne_formatting_sample_title_authors_affiliations.pdf>

Thank you very much for your comment. We have now revised the manuscript style and file names as per PLOS ONE guide to make it compliant with PLOS ONE's style requirements.

2. We note that the grant information you provided in the ‘Funding Information’ and ‘Financial Disclosure’ sections do not match.

When you resubmit, please ensure that you provide the correct grant numbers for the awards you received for your study in the ‘Funding Information’ section.

Thank you very much for your comment. Apologies for an oversight we have now updated the grant information and have provided with the correct details.

“The review was funded by National Institute for Health Research as part of feasibility randomised control trial under scheme Research for Patient Benefit (RfPB). Funding award ID: NIHR201146. The funders had no role in study design, data collection and analysis, decision to publish, or preparation of the manuscript.”

3. Thank you for stating the following in the Acknowledgments Section of your manuscript:

The review was funded by National Institute for Health Research as part of feasibility randomised control trial titles “Does knowledge of liver fibrosis affect high risk drinking behaviour (KLIFAD)? A feasibility randomised controlled trial”. Funding award ID: NIHR201146. JRM and HK receive salary support from a Medical Research Council Clinician Scientist Fellowship [grant number MR/P008348/1]

We note that you have provided additional information within the Acknowledgements Section that is not currently declared in your Funding Statement. Please note that funding information should not appear in the Acknowledgments section or other areas of your manuscript. We will only publish funding information present in the Funding Statement section of the online submission form. 
Please remove any funding-related text from the manuscript and let us know how you would like to update your Funding Statement. Currently, your Funding Statement reads as follows:

The funders had no role in study design, data collection and analysis, decision to publish, or preparation of the manuscript

Please include your amended statements within your cover letter; we will change the online submission form on your behalf.

Thank you very much for your comment. We have now removed funding information from acknowledgment section and from manuscript.

Please add following statement in funding section of online submission form.

“The review was funded by National Institute for Health Research as part of feasibility randomised control trial under scheme Research for Patient Benefit (RfPB). Funding award ID: NIHR201146. The funders had no role in study design, data collection and analysis, decision to publish, or preparation of the manuscript.”

Please add following statement in acknowledgement section of online submission form.

“Alison Ashmore, senior research librarian (Nottingham University Libraries) contributed to finalising the search strategy.”

4. Thank you for stating the following in your Competing Interests section:

NO authors have competing interests

Please complete your Competing Interests on the online submission form to state any Competing Interests. If you have no competing interests, please state "The authors have declared that no competing interests exist.", as detailed online in our guide for authors at <http://journals.plos.org/plosone/s/submit-now> 
 This information should be included in your cover letter; we will change the online submission form on your behalf.

Thank you very much for your comment. We will be grateful for updating the online submission form on our behalf. Please add following statement

"The authors have declared that no competing interests exist."

5. We note that Figure 1 in your submission contain [map/satellite] images which may be copyrighted. All PLOS content is published under the Creative Commons Attribution License (CC BY 4.0), which means that the manuscript, images, and Supporting Information files will be freely available online, and any third party is permitted to access, download, copy, distribute, and use these materials in any way, even commercially, with proper attribution. For these reasons, we cannot publish previously copyrighted maps or satellite images created using proprietary data, such as Google software (Google Maps, Street View, and Earth). For more information, see our copyright guidelines: <http://journals.plos.org/plosone/s/licenses-and-copyright>.
 
We require you to either (1) present written permission from the copyright holder to publish these figures specifically under the CC BY 4.0 license, or (2) remove the figures from your submission:
 
a. You may seek permission from the original copyright holder of Figure 1 to publish the content specifically under the CC BY 4.0 license.  
 
We recommend that you contact the original copyright holder with the Content Permission Form (<http://journals.plos.org/plosone/s/file?id=7c09/content-permission-form.pdf>) and the following text:
“I request permission for the open-access journal PLOS ONE to publish XXX under the Creative Commons Attribution License (CCAL) CC BY 4.0 (<http://creativecommons.org/licenses/by/4.0/>). Please be aware that this license allows unrestricted use and distribution, even commercially, by third parties. Please reply and provide explicit written permission to publish XXX under a CC BY license and complete the attached form.”
 
Please upload the completed Content Permission Form or other proof of granted permissions as an "Other" file with your submission.
 
In the figure caption of the copyrighted figure, please include the following text: “Reprinted from [ref] under a CC BY license, with permission from [name of publisher], original copyright [original copyright year].”
 
b. If you are unable to obtain permission from the original copyright holder to publish these figures under the CC BY 4.0 license or if the copyright holder’s requirements are incompatible with the CC BY 4.0 license, please either i) remove the figure or ii) supply a replacement figure that complies with the CC BY 4.0 license. Please check copyright information on all replacement figures and update the figure caption with source information. If applicable, please specify in the figure caption text when a figure is similar but not identical to the original image and is therefore for illustrative purposes only.
The following resources for replacing copyrighted map figures may be helpful:
 
USGS National Map Viewer (public domain): <http://viewer.nationalmap.gov/viewer/>
The Gateway to Astronaut Photography of Earth (public domain): <http://eol.jsc.nasa.gov/sseop/clickmap/>
Maps at the CIA (public domain): <https://www.cia.gov/library/publications/the-world-factbook/index.html> and <https://www.cia.gov/library/publications/cia-maps-publications/index.html>
NASA Earth Observatory (public domain): <http://earthobservatory.nasa.gov/>
Landsat: <http://landsat.visibleearth.nasa.gov/>
USGS EROS (Earth Resources Observatory and Science (EROS) Center) (public domain): [http://eros.usgs.gov/#](http://eros.usgs.gov/)
Natural Earth (public domain): <http://www.naturalearthdata.com/>

Thank you very much for your comment. Thanks for highlighting the issue. We have now removed these figures from supplementary material.

6. Please include your tables as part of your main manuscript and remove the individual files. Please note that supplementary tables (should remain/ be uploaded) as separate "supporting information" files.

Thank you very much for your comment. We have now provided the tables as part of main manuscript and have uploaded supplementary material as separate files titled " S1 Table, S2 Table, S3 Table, and S4 Table "

Reviewers' comments:

Review Comments to the Author

Please use the space provided to explain your answers to the questions above. You may also include additional comments for the author, including concerns about dual publication, research ethics, or publication ethics. (Please upload your review as an attachment if it exceeds 20,000 characters)

**Reviewer #1:**

This review is informative and helps the reader gain an easy understanding about the process of alcohol recovery, as well as the importance of incorporating narratives.

Thank you very much for your comment. We hope the article will attract similar attention from the wider audience of the journal.

It may be helpful if the authors can add more details about the quality analysis of the studies reviewed. While the use if critical appraisal skills program has been mentioned, for the reader who may not be aware of this tool, a few additional details will help give a clearer picture as to how the quality of the study was assessed.

Thank you very much for your comment. We have now provided details on quality analysis of included studies as advised by the reviewer. We have included following paragraph in method section of study.

“Quality assessment of qualitative evidence synthesis has been a matter of debate for many decades (1). Cochrane Qualitative and Implementation Methods Group recommendations are to use a tool that takes the multi-dimensional concept of qualitative evidence into account (1).

Keeping this in view, the quality of included studies and risk of bias was assessed using the Critical Appraisals Skills Programme (CASP) tool for qualitative research (2). The CASP tool focuses on three domains, study design, results validity, and generalisability. Each domain has a set of questions. Based on the response to these questions the studies were marked as low, medium, or high quality. The studies which provided satisfactory information in all domains were marked as high quality, with missing or unsatisfactory information in one domain as medium quality, and with missing or unsatisfactory information in two or more domains as low quality.”

**Reviewer #2:**

This systematic review is an excellent and balanced overview that includes 32 studies (29 qualitative, 3 mixed-methods, 1055 sample size from Europe and United states, includes multiple databases, spanning 30 years (male predominant, 41 % from AA recovery group), gives an interesting perspective of role of recovery narratives in alcohol recovery. It aims to produce a conceptual framework with multiple dimensions describing the characteristics of alcohol misuse recovery narratives from the available literature. It focuses mostly on Alcoholic Anonymous(AA) or other unstructured formal treatment for alcohol use recovery. It has a good subgroup analysis of all dimensions. Major limitation is, its missing people in lower income countries, as no research literature available in those areas, lacks information who have recovered outside of main services or those who have used services other than AA, and lacks data on females population and ethnic diversity especially in non Caucasian. It does include indigenous population and LGBTQ. Furthermore, this framework will help to identify gaps in knowledge, summarize range of methods, enables developing future research.

They have registered in Prospero, followed Prisma guidelines, is part of NIH grant, did CASP for ROB assessment, Has good Inclusion, exclusion criteria, and approach towards outcomes.

Thank you very much for your appreciation and taking time to review the manuscript.

However, I have a suggestions that could be added to the article.

In my opinion, Tabulating all of these dimension definitions in a excel sheet with listing the articles or adding this information to the attached excel sheet would Make it more easy to follow. It will help to improve the flow and readability of the text.

Thank you very much for your comment. The detail of individual references with study ID and reference number has been provided in Supporting S2 Table. We have now added S4 Table in supporting information including the references and explanation of individual dimensions.

If a further subgroup analysis could be added separately to include information on concomitant substance use disorders(drug use) and/or mental health issues, then it would give a good perspective in terms forming a better framework. I recommend the article be published.

Thank you very much for your comment. We have now added additional subgroup analysis describing dual diagnosis aspect of alcohol misuse and mental health. The data was insufficient to have sperate subgroup analysis on polydrug use. We have added this as a limitation of the review. The additional subgroup analysis reads as

**Alcohol and mental health:** Analysis of studies discussing dual diagnosis of alcohol misuse and mental health problems showed participants often suffered with negative self-perceptions, including low self-esteem, lack of love from others, lack of desire to belong, anger, and shame (3-11). Mental health problems often acted as a trigger to drink harmfully (5). Common mental health problems reported were anxiety, depression, obsessive compulsive disorders, post traumatic disorders (mostly due to difficult childhoods), attention seeking behaviours, eating disorders, and emotional instability (3-7, 9). Facilitators to recovery were integrated support from mental health and substance misuse services, a flexible and trustworthy relationship with care providers, individualised treatment pathways, and easy to understand step-by-step support. Whereas barriers to recovery were undiagnosed or unrecognised mental health problems, inadequate support from mental health services, underfunded services, and punitive response to alcohol misuse (9). Participants who had negative recovery experience reported ongoing mental health difficulties comprised of anxiety, depression, and intrusive or disturbing memories. This in turn impacted longevity of sobriety (11).

**Reviewer #4:**

Nicely written article which identifies a conceptual framework including various aspects of chronic alcohol abuse and dependence, the narrative summarize the characteristics well, more than 1000 patient included and psychosocial aspects were explored which adds value.

Thank you very much for your comment. We hope the article will attract similar attention from the wider audience of the journal.

**Reviewer #6:**

This paper aims to produce a conceptual framework describing the characteristics of alcohol misuse recovery narratives that are in the research literature, to inform the development of research, policy, and practice. The systematic review and data synthesis were well organized and all the elements needed for this type of quantitative research appear to be in place. Much of the tabular information is well summarized in the appendices.

The review may have provided characteristics of alcohol recovery narratives, with implications for both research and healthcare practice. It demonstrated knowledge gaps in relation to alcohol recovery narratives of people living in lower income countries, or those who recovered outside
of mainstream services.

Thank you very much for your comment and providing the feedback.

Although the information is descriptive and quantitative, the risk of bias from each study should have been represented in tabular form. This is lacking in Table 1 of the supplemental material. Some comments concerning possible sources of heterogeneity and publication bias should have been addressed, especially with the geographical distribution of the data.

Thank you very much for your comment. Sorry for not providing the specific details, we have now added supporting S3 table describing risk of bias and quality assessment for individual studies.

For reviewer convenience we have also included table at end of this document.

Thank you again for your time and consideration.

Yours Sincerely,

Dr Mohsan Subhani

MBBS, MRCP Medicine, MRCP Gastroenterology

Nottingham Digestive Diseases Biomedical Research Centre (NDDC)

University of Nottingham UK

S3 Table: Risk of bias and Quality of included studies

| **Stud ID** | **Study quality** | **Was there a clear statement of the aims of the research?** | **Is a qualitative methodology appropriate?** | **Was the research design appropriate to address the aims of the research?** | **Was the recruitment strategy appropriate to the aims of the research?** | **Was the data collected in a way that addressed the research issue?** | **Has the relationship between researcher and participants been adequately considered?** |
| --- | --- | --- | --- | --- | --- | --- | --- |
| Best et al.(2016) | Low | Yes | N/A | Yes | Yes | Yes | No |
| Burman et al. (1997) | Medium | Yes | Yes | Yes | Yes | Yes | No |
| Cain et al. (1991) | Low | Yes | Yes | Poorly defined data analysis | Yes | Yes | No |
| Christensen and Elmeland. (2015) | Medium | Yes | Yes | Poorly defined data analysis | Yes | Yes | No |
| Dalgarno et al.(2018) | Medium | Yes | Yes | Yes | Yes | Yes | No |
| Dunlop and Tracy.(2013) | High | Yes | Yes | Yes | Yes | Yes | Yes |
| Dunlop and Tracy.(2013) | High | Yes | Yes | Yes | Yes | Yes | Yes |
| Garland et al.(2012) | Medium | Yes | Yes | Yes | Yes | Yes | No |
| Gubi and Marsden-Hughes.(2013) | Medium | Yes | Yes | Yes | Yes | Yes | No |
| Haarni and Hautamäki. (2010) | Medium | Yes | Yes | Yes | Yes | Yes | No |
| Hanninen and Koski-Jannes.(1999) | Medium | Yes | Yes | Yes | Yes | Yes | No |
| Inman and Kornegay.(2004) | Medium | Yes | Yes | Yes | Yes | Yes | No |
| Jones et al.(2013) | Medium | Yes | Yes | Yes | Yes | Yes | No |
| Laitman and Lederman.(2008) | Low | Yes | Yes | Yes | Yes | Can't tell | No |
| Laville et al. (2006) | Low | No | Yes | Yes | Yes | Yes | N/A |
| Lederman and Menegatos.(2011) | Medium | Yes | Yes | Yes | Yes | Yes | No |
| Liezille Jacobs. (2015) | Medium | Yes | Yes | Yes | Yes | Yes | No |
| Mohatt et al.(2008) | Low | Yes | No | Yes | Yes | Yes | No |
| Mohatt et al.(2008) | Medium | Yes | Yes | Yes | Yes | Yes | No |
| Newton et al.(2007) | Medium | Yes | Yes | Yes | Yes | Yes | No |
| Opačić et al. (2019) | High | Yes | Yes | Yes | Yes | Yes | Yes |
| Paris and Bradley.(2001) | High | Yes | Yes | Yes | Yes | Yes | Yes |
| Punzi and Tidefors. (2014 ) | Medium | Yes | Yes | Yes | YEs | Yes | No |
| Robbins et al. (2015) | Medium | Yes | Yes | Yes | Yes | Yes | No |
| Rowan and Butler. (2014) | High | Yes | Yes | Yes | Yes | Yes | Yes |
| Sawer et al. (2020) | Medium | Yes | Yes | Yes | Yes | Yes | No |
| Stott and Priest. (2018) | Medium | Yes | Yes | Yes | Yes | Yes | No |
| Strobbe and Kurtz.(2012) | Low | Yes | Yes | Yes | Yes | Can't tell | No |
| Suprina et al. (2006) | Medium | Yes | Yes | Yes | Yes | Yes | No |
| Vaughn and Long. (1999) | Medium | Yes | Yes | Yes | Yes | No | No |
| Weegmann and Piwowoz-Hjort.(2009) | Medium | Yes | Yes | Yes | Yes | Yes | No |
| Zakrzewski and Hector. (2004) | High | Yes | Yes | Yes | Yes | Yes | Yes |
